# A Typology of Emerging Market SMEs’ COVID‐19 Response Strategies: The Role of TMTs and Organizational Design

**DOI:** 10.1111/1467-8551.12591

**Published:** 2022-02-16

**Authors:** Pushyarag Puthusserry, Timothy King, Kristel Miller, Zaheer Khan

**Affiliations:** ^1^ Kent Business School University of Kent Canterbury CT2 7NZ UK; ^2^ Ulster University Business School University of Ulster Belfast BT15 1ED UK; ^3^ University of Aberdeen Business School University of Aberdeen Aberdeen AB24 3FX UK; ^4^ InnoLab University of Vaasa Vaasa 65200 Finland

## Abstract

The unique challenges posed by COVID‐19 call for new insights into how firms respond to multiheaded and multistage evolving global crises. Whilst prior research acknowledges the potential role flexible organizational designs and top management teams (TMTs) have for crisis management, these bodies of literature have evolved separately with limited cross‐fertilization. In this study, we seek to provide a contextualized explanation of research phenomena by drawing upon multiple layers of context – namely the environment, TMT and organisational context. Our findings provide vital insights into how emerging market Indian SMEs’ organizational designs and TMT configurations led to differential COVID‐19 crisis response strategies. We develop a typology that identifies four strategic responses and illustrate that not all emerging market SMEs are vulnerable at the time of crisis. Our findings extend knowledge on how emerging market SMEs can navigate external shocks such as those caused by COVID‐19. In particular, our research has implications for policymakers and emerging market firms seeking to understand and implement effective organizational designs and policies that can weather the current COVID‐19 pandemic, as well as future multiheaded and multistage black swan crises.

## Introduction

Crises originating externally can threaten firms’ operations, performance and survival (Chau *et al*., 2012; Wenzel, Stanske and Lieberman, 2020; Whiteman and Cooper, 2011). Such crises can be considered black swan events when they occur with ‘low probability [but have] high impact [and are] perceived by critical stakeholders to threaten the viability of the organization’ (Pearson and Clair, 1998: 66). COVID‐19 represents such a crisis (Winston, 2020). Yet, COVID‐19 differs from previous crises and black swan events in that it presents both a three‐pronged and a multistage challenge for firms: ‘the virus, the panic about the virus and the business and economic implications’ (The Australian Institute of Company Directors, 2020).

The complex multiheaded and multistage nature of the pandemic has forced top management teams (TMTs)^1^ to make decisions in real time (Caligiuri *et al*., 2020), which includes flexing organizational designs. Ultimately, COVID‐19 emphasizes the point that organizations need to do things differently when faced with black swan events (Winston, 2020). This is evident by the fact that COVID‐19 has completely disrupted business models, supply chains, internal operations and societal structures and norms on a global scale. It has also accelerated the adoption of new technologies and new ways of doing business (Altman, 2021).

Consistent with upper echelons theory (UET) (Hambrick, 2007; Hambrick and Mason, 1984), it could be suggested that firm responses to a crisis are governed by bounded rationality (Cohen, Bingham and Hallen, 2019) and by the breadth and depth of managers’ characteristics and experiences, which influence strategic decision‐making and use of firm resources (Haynes and Hillman, 2010; Neely *et al*., 2020; Puthusserry *et al*., 2021). TMTs also make strategic decisions regarding organizational design, which encapsulates how organizations should be structured to function effectively and efficiently (Burton and Obel, 2018; De Massis, Eddleston and Rovelli, 2020). For instance, it is suggested that internal organizational design factors are key determinants of organizational responses to crises (Bundy *et al*., 2017; George, Lakhani and Puranam, 2020) and can help to explain the ability of organizations to internally adapt and respond to external challenges (Doern, Williams and Vorley, 2019; Williams *et al*., 2017). It is suggested that firms’ organizational designs should be malleable in order to adapt to internal and external contingencies, which can be both organizational and environmental (Clement and Purnanam, 2018). Yet, the combined role of TMTs and organizational design as both determinants and explanatory concepts to understand firms’ responses to different crisis contexts has been underexplored (Bundy *et al*., 2017; George, Lakhani and Puranam, 2020; Junni *et al*., 2013; O'Reilly and Tushman, 2013).

In this paper, we align with scholars who stress the importance of a contextualized explanation of research phenomena (Paavilainen‐Mäntymäki *et al*., 2020; Plakoyiannaki and Budhwar, 2021; Welch *et al*., 2011). In order to develop a more comprehensive understanding of the contextual influence on Indian digital SMEs’ COVID‐19 response strategies, we adopt a multilayered approach of investigating the environment, organization and TMT‐level contexts (Autio *et al*., 2014; Shepherd and Rudd, 2014). In line with Hitt *et al*. (2007), we believe that the multilayered approach can help explain the behaviours of TMTs and organizations in addressing challenges posed by the global COVID‐19 crisis. First, we suggest that COVID‐19 presents a unique overlaying environmental context, where prior scholars identify the need to better understand how firms respond to black swan crises (e.g. Ahlstrom *et al*., 2021; Amankwah‐Amoah, Khan and Wood, 2021). Second, we study the context in terms of the impact of COVID‐19 on emerging market Indian digital SMEs, combining the environmental and organizational context. Prior research suggests that crises may be more pronounced for emerging market SMEs (Albonico, Mladenov and Sharma, 2020; Penrose, 2000). More specifically, SMEs originating from weaker institutional environments, typical of emerging markets such as India, often face greater resource‐based weaknesses and innate institutional voids (Cowling, Liu and Zhang, 2020; Doh, Tashman and Benischke, 2019; Khanna and Palepu, 1997), which could be suggested to make them more reliant on internal capabilities and resources during crises. Hence, we focus on the potential role of TMTs and organizational structure and coordination mechanisms which represent the organizational and TMT contexts, respectively (Shepherd and Rudd, 2014).

Insights gained from studying previous crises, such as the global financial crisis of 2008–2009, and shocks from natural disasters, technological advancement and political turbulence are informative to aid future organizational planning and resilience (Doern, Williams and Vorley, 2019; Williams *et al*., 2017). However, much remains unknown about how firms respond to black swan crises (e.g. Ahlstrom *et al*., 2021; Amankwah‐Amoah, Khan and Wood, 2021). This goes beyond building collective capacities and skills to exploring how firms’ internal organizing practices and resource endowments condition responses. Consequently, we suggest that investigating the influence of TMTs and organizational design on emerging market SMEs’ responses to COVID‐19 will help with the verification of pre‐existing theoretical reasoning in specific crisis contexts, and/or may lead to theory refinement, elaboration or extension through adding contextualized contingency perspectives to longstanding theoretical insights. To achieve this, we consider the following interrelated research questions: (1) How do internal and external contingencies of COVID‐19 influence emerging market SMEs? (2) How do organizational design and board capital shape the strategic actions taken to overcome COVID‐19 contingencies?

Drawing on contextualized explanations, our findings provide insights into how emerging market Indian SMEs’ organizational designs and TMT configurations led to differential COVID‐19 crisis response strategies. This responds to calls for research investigating how firms have responded to COVID‐19 (Budwar and Cumming, 2020) and for more contextualized insights into strategy making in times of crisis (Wenzel, Stanske and Lieberman, 2020). We contribute to theory refinement and expansion of the role of TMTs and organizational design during different crisis contexts, through the presentation of a typology, which identifies four response strategies (*reactive*, *proactive*, *conforming* and *innovative*) that emerging market SMEs may demonstrate during the early stages of a black swan crisis such as COVID‐19. Our empirical insights expand research which suggests that not all SMEs, and indeed emerging market SMEs, are vulnerable during crises. Instead, we emphasize that emerging market SMEs can counteract the evolving and multifaceted challenges COVID‐19 presents through market penetration, product development and diversification growth strategies during times of crisis.

## Theoretical background

### Crisis response strategies

Careful crisis management is fundamental to minimize the effects of crises on organizations (Dowell, Shackell and Stuart, 2011). Employed successfully, it can facilitate the quick restoration of disrupted or weakened systems (Williams *et al*., 2017) and be a source of competitive advantage for those able to respond quickly to exploit new emerging opportunities (Bundy *et al*., 2017). Despite an established body of literature retrospectively exploring the aftermath of crisis events or analysing a crisis as a process, which unfolds over time (Claeys and Cauberghe, 2014; Williams *et al*., 2017), scarce research has explored a crisis as an event which occurs suddenly, is unexpected and has continued longevity and uncertainty (Merendino and Sarens, 2020).

Prior literature provides some guidance on how firms may react and respond to crises. For example, Williams *et al*. (2017) evaluate existing crisis management and resilience literature to identify how different factors such as particular leadership styles, crisis management teams and diverse capability endowments impact firms’ actions and responses during crises. Wenzel, Stanske and Lieberman (2020) also synthesize existing research on crises to identify four potential strategic responses, namely, retrenchment, persevering, innovating and exit. Theoretical frameworks like these are useful to generate managerial insights, while learning from prior failures and challenges can aid future crisis preparedness (Carmeli and Schaubroeck, 2008). However, Foss (2020) stresses that caution is needed when interpreting prior crisis management research; specifically, they caution that ex‐ante predictions can yield incomplete insights into the intricacies of strategizing during unique contexts, especially during heightened levels of uncertainty which COVID‐19 presents. Thus, we align with scholars who stress the importance of contextualized explanations in order to aid theory refinement, verification, elaboration and extension across different contexts (Eriksson and Engstrom, 2021; Paavilainen‐Mäntymäki *et al*., 2020; Welch *et al*., 2011) and consider the influence that environmental, organizational and TMT contexts can have on an organization's strategizing and decision‐making under prolonged external shocks.

Different contexts have been suggested to lead to varying ‘contingencies’; both internal and external to organizations (Sharma *et al*., 2020). Contingencies are not only determined by firm size, lifecycle stage and external market and institutional factors (Miller *et al*., 2021), but also by the nature and magnitude of the crisis, which may pose unique threats, challenges and opportunities (Foss, 2020; Williams *et al*., 2017). Consequently, scholars suggest the need to explore how a change in context may lead to similar or different outcomes of pre‐existing theoretical reasoning (Eriksson and Engstrom, 2021; Foss, 2020). Much crisis‐related research is in the context of developed market firms, where institutional support can be substantial in helping firms to respond to external shocks – such as for example, the bailout support US automotive manufacturers received to deal with the impact of the 2007–2009 global financial crisis (Congleton, 2009). However, there has been relatively little research in the context of emerging market firms’ response strategies to external crisis – who are unlikely to benefit from the strong institutional support developed market firms may receive. Consequently, it is important to examine emerging market firms’ response strategies in order to provide contextualized explanations for this important topic.

To develop an effective crisis response strategy to a crisis such as COVID‐19, organizations must possess not only the necessary capabilities needed to rapidly reconfigure and leverage resources to adapt to sudden changes in the environment (Foss, 2020), but also the flexibility to readjust basic assumptions, behaviours (Pearson and Clair, 1998), routines and processes and ensure emotional regulation (Merendino and Sarens, 2020). The socio‐economic shock created by COVID‐19 resulted in the need for new work‐from‐home (WFH) business models enforced by governments since March 2020. This required organization to undertake rapid transformations to their organizational designs (Seidl and Whittington, 2020; Spicer, 2020), requiring organizational flexibility. We argue that critical determinants of organizational flexibility are contingencies inherent in both organizational (internal) and environmental (external) contexts (Schreyögg and Sydow, 2010). We proffer that this nexus of internal and external alignment can be better understood by studying the combinative effect of organizational design and resources and capabilities inherent within the TMT and board. These two streams of literature have so far evolved separately with little cross‐fertilization.

### A contingency view of organizational design

Organizational design encapsulates the structures of accountability and responsibility created to develop and implement strategies, practices and processes that facilitate organizational structure (De Massis, Eddleston and Rovelli, 2020; Greenwood and Miller, 2010). When disruptions occur, the organizational design could be an important element that shapes organizational responses. However, research to date is inconclusive on how design has impacted organizations’ responses to different crisis contexts (Foss, 2020; George, Lakhani and Puranam, 2020).

It can be challenging to deconstruct core components of organizational design, given the complex webs of interactions, coordination and effective structures needed to ensure internal and external fit (cf. Clement and Puranam, 2018; Puranam, Alexy and Reitzig, 2014). However, Burton, Obel and Håkonsson (2020) suggest that it can be delineated into structure and coordination, where an organization should seek congruence. They suggest that organizational structure involves delineating larger problems or purposes into subunits, with each subunit having autonomy over task assignment and resources. Coordination is a differentiated but complementary function linking these subunits and smaller tasks to efficiently realize strategic objectives. Coordination also involves control mechanisms, decisions, information sharing and incentive systems (Burton and Obel, 2018; Burton, Obel and Håkonsson, 2020).

In seeking to understand the role of organizational design in crisis response strategies by different types of firms, we draw upon key insights from structural contingency theory (Pennings, 1987), which builds on general contingency theory and suggests there is no ‘one best way’ of organizing (e.g. Mintzberg, 1989). Van de Ven, Ganco and Hinings (2013) suggest that organizations are a constrained optimization, requiring appropriate levels of differentiation and integration to address contingencies. These contingencies can vary and include both internal and external contingencies where both are intertwined (Pennings, 1987). For example, company size and lifecycle may dictate the number of subunits and coordination mechanisms, whereas environmental contingencies could take the form of stable versus unstable market structures. Prior research suggests that these uncertainties force organizations to create discrete structures of organizational units, which are malleable and can be (re)configured in different ways to deal with different tasks (Foss, 2020). Burton and Obel's (2004, 2018) multi‐contingency perspective complements structural contingency theory to introduce multiple dimensions which impact organizational design, such as the influence of TMTs, characterized by heterogeneous human and social capital dimensions. However, the traditional contingency literature has paid insufficient attention to the interactions and role of TMTs in responding to prolonged external shocks (Burton and Obel, 2018; De Massis, Eddleston and Rovelli, 2020). In this paper, we examine these components by integrating insights from the literature on TMTs and boards.

### The combinative strategic role of board and TMT, and organizational design

The roles played by a strategic leadership team in determining situational awareness and effective strategy design and implementation during a crisis are widely acknowledged (Campbell and Sinclair, 2009; Dowell, Shackell and Stuart, 2011; Francis, Hasan and Wu, 2012). A firm's strategic leadership team can be delineated into the TMT – including influential executives such as the Chief Executive Officer (CEO) and the board of directors – who all play distinct but integrated roles, which coalesce to ensure the smooth running and competitive position of the organization. A substantial literature has explored their roles in organizational decision‐making and actions (Haynes and Hillman, 2010; Lewin and Stephens, 1994; Lubatkin *et al*., 2006). Whilst much of this research has focused on boards’ monitoring (and advising) roles, less attention has been paid to resource provision (e.g. Hillman, Nicholson and Shropshire, 2008; Hillman, Withers and Collins, 2009), which is important during a crisis, and particularly so for emerging market SMEs, given that these firms receive limited support from local institutions. Yet, their roles in helping emerging market SMEs respond to exogenous, multifaceted, multistage and black swan crises have not received much attention.

The role of boards and TMTs has its foundation within UET, which suggests that organizations reflect their TMTs (Hambrick and Mason, 1984). Executive and non‐executive board members’ human (expertise, experience and education) and social capital (networks and reputation) constitute the breadth of board capital (Haynes and Hillman, 2010; Zorn *et al*., 2017). In contrast, their embeddedness within the focal industry (e.g. managerial positions, experience and/or interlocking directorships) constitutes the depth of board capital. Holistically, board capital represents significant resources, which can be determinants of organization strategizing and success (Dalton *et al*., 1998; Lungeanu and Zajac, 2019). Despite this, existing research is agnostic over what mix of human and social capital amongst TMTs and boards may be optimal across different contexts.

Furthermore, existing research provides mixed findings on the need for TMT and board diversity. For instance, Keck (1997) suggests that in stable but potentially turbulent industry contexts, homogeneous TMTs may be most effective. In contrast, in dynamic and complex industries, heterogeneous and short‐tenured boards are more strategically effective. De Villiers, Naiker and Van Staden (2011) suggest that independent board directors with breadth of experience may be more valuable during periods of turbulence because they are more likely to ask challenging questions, which protects stakeholders. However, Merendino and Sarens (2020) suggest that boards can remain passive during crises, thereby undermining their effectiveness. Other research suggests that during uncertain industry environments, boards and TMTs have greater strategic discretion (Finkelstein and Hambrick, 1990; Haleblian and Finkelstein, 1993). Indeed, Carpenter and Fredrickson (2001) draw upon Mischel's (1977) concept of ‘situation strength’, where it could be suggested that environmental forces dictate the need for particular TMT characteristics and actions – akin to structural contingency theory. Therefore, it could be suggested that during a crisis, positive (or negative) impacts of board diversity may be accentuated, implying a need for a contingency perspective for board composition. For example, although larger boards may have greater depth and breadth of directors’ human and social capital to draw upon, whereas smaller boards may prove more agile in responding to immediate threats to firm survival (Dowell, Shackell and Stuart, 2011). However, there is a lack of empirical research exploring how TMT dynamics may influence strategies and actions in different and turbulent environmental contexts such as COVID‐19 (cf. Amankwah‐Amoah, Khan and Osabutey, 2021; Collings *et al*., 2021).

The development of a board is a resource endowment, where its configuration and reconfiguration over time is a key element of organizational design (Mangena, Tauringana and Chamisa, 2012; Merendino and Sarens, 2020). Furthermore, it is suggested that the factors impacting organizations in turn are impacted by an organization's structure (Child, 1972). These include the role of leadership, fulfilled by the board of directors and key firm executives (Child, 1972; De Massis and Rondi, 2020; Mintzberg, 1989). Whilst prior research on UET identifies the influence of industry‐level changes during a crisis, much less is known on micro‐level issues such as how boards and TMTs respond to crises (Merendino and Sarens, 2020; Mumford *et al*., 2007; Verbeke and Yuan, 2020). Furthermore, limited UET research to date has studied the influence of TMTs on organizational design (Lewin and Stephens, 1994; Rovelli *et al*., 2020) and how they may affect firms’ responses to extreme black swan events.

From the literature, it can be suggested that organizations with more malleable and flexible organizational designs are associated with more resilience and innovative responses to external shocks (e.g. Foss, 2020; Hitt, Keats and DeMarie, 1998; Merendino and Saren, 2020). However, a firm's ability to enact structural design contingency is impacted by its TMT and board of directors and its resources and capabilities, often typified by organizational size (Zuzul and Tripsas, 2020). For example, large firms generally have more diverse human capital stock, formalized structures and reporting mechanisms, which may create internal coordination challenges that could make it difficult to adapt and respond to external uncertainties quickly (Nguyen and Bryant, 2004; Reeves, Lang and Carlsson‐Szlezak, 2020). Larger organizations are also associated with greater decentralization and a tendency to adopt incentives and formalization mechanisms to foster managerial commitment and help shape decision‐making (Alonso, Dessein and Matouschek, 2008; De Massis, Eddleston and Rovelli, 2020; Mintzberg, 1979). In contrast, smaller firms typically have smaller TMTs and boards comprising internal executives and amorphous organizational designs – characteristic of flat organizational structures. Amorphous organizational designs have been found to enable strategic flexibility, agility and authority, which helps quickly address external challenges (Zuzul and Tripsas, 2020), useful during severe crises. In other words, it could be suggested that directors and/or CEOs of small firms centralize decision‐making authority or make decisions autonomously, which mitigates coordination, communication and/or formalization challenges (De Massis, Eddleston and Rovelli, 2020; Ling *et al*., 2008). However, small firms with less formalized and more fragmented structures and with limited human resources may, conversely, be less resilient to market shocks (Lai *et al*., 2016; Storey and Skyes, 1996).

In seeking to refine and aid contextualized explanation of the existing TMT and organizational design literature, we proffer that strategic flexibility can be achieved partly through structural, human and coordination elements of organizational design, which facilitates recombining and reconfiguring organizational resources stocks rapidly (Amankwah‐Amoah, Khan and Osabutey, 2021; Teece, Pisano and Shuen, 1997) and helps overcome organizational inertia (Zhou and Wu, 2010). Strategic flexibility is also reliant upon fast execution of decision‐making and the capability of senior executives and boards who control and configure the structure, including task specialization, bundling and sequencing (George, Lakhani and Puranam, 2020; Williams *et al*., 2017). Thus, strategic flexibility is reliant upon both structural and coordination flexibilities (Buton and Obel, 2018; Zhou and Wu, 2010), which we argue could be affected by both organizational design and board capital as well as the dynamics between them. More specifically, we assert that flexible organizational design and the depth and breadth of TMTs’ human and social capital should be important factors affecting firms’ strategic responses to COVID‐19‐related challenges and opportunities.

## Context and methods

Due to the nascent nature of COVID‐19, a qualitative case study methodology was deemed suitable to generate a detailed contextual description of the impact of the phenomenon on firms (Lincoln and Guba, 1985; Yin, 2018). We follow scholars such as Paavilainen‐Mäntymäki *et al*. (2020), who suggest that qualitative research can be useful for explaining context and, in particular, how ‘generative mechanisms’ can explain how observable events occur. This relates to the concept of ‘contextualized explanation’ (Plakoyiannaki and Budhwar, 2021; Welch *et al*., 2011), which can help understand the ‘why and how’ in particular contexts and can aid theory refinement, elaboration or extension (Paavilainen‐Mäntymäki *et al*., 2020; Plakoyiannaki and Budhwar, 2021; Welch *et al*., 2011).

Many scholars advocate the importance of understanding the context in which events occur as being critical to understanding variances in behaviours or outcomes generated (Eriksson and Engstrom, 2021; Welch *et al*., 2011). A crisis presents a significant change to the environmental context of an organization, where scholars stress the need to integrate context into organizational studies (e.g. Eriksson and Engstrom, 2021; Foss, 2020; Prime, Obadia and Vida, 2009). As noted earlier, this research context is multilayered, exploring the environmental context of COVID‐19 within the organizational context of emerging market Indian digital SMEs and their TMTs (Autio *et al*., 2014; Shepherd and Rudd, 2014).

### Research background: Indian digital SMEs

India is the global hub of IT (information technology) and ITeS (information technology‐enabled services), with a market share of 55% of the $200–250 bn global services sourcing business in 2019–2020 (NASSCOM, 2017; Parkin and Waters, 2021). The core competencies of Indian IT, such as highly skilled, low‐cost workers and well‐developed IT infrastructure and policies, have encouraged multinational enterprises (MNEs) worldwide to delegate their critical functions to India (IBEF, 2021). The sector accounted for 8% of Indian gross domestic product in 2020 (IBEF, 2021), and it generates around $180 bn in revenue a year (NASSCOM, 2017). However, COVID‐19 and the subsequent economic crisis have resulted in a decline in the industry growth rate (3.6% in the first half of 2020 compared to the first half of 2019) as firms had to reduce their IT spending (IDC, 2020). The IDC (2020) report also indicates that the new normal situations have created opportunities for firms in newer technology domains such as collaborative applications, application platforms, security software, system and service management software and content workflow and management applications. These new opportunities have shown an increase in IT spending by the small and medium‐sized business segment, cloud‐native software start‐ups and government initiatives for data localization. We aim to study the influence of this external market context on Indian digital firms and how they leveraged their internal organizational and TMT human and social capital to navigate through such a context.

### Data collection

To answer our research questions, a theory‐based sampling strategy was adopted (Corbin and Strauss, 2008) comprising firms that were affected by COVID‐19. We targeted emerging market SMEs,^2^ which are typically more vulnerable during a crisis than developed market or large firms due to receiving limited support from formal institutions. Due to the relatively limited research on this topic and considering that firms had only experienced the first 4 months of the onset of COVID‐19 when this research was conducted, contextual explanatory reasoning was sought to understand these firms’ COVID‐19 response strategies. Therefore, we used ‘polar cases’ to study if there were divergent behaviours and patterns of responses based on opposing characteristics (Elsbach and Kramer, 2003). Consequently, eight Indian digital firms were chosen of varying sizes, ages and exhibiting the diversity of TMT structure. The sample consisted of firms from the micro (n = 3), small (n = 2) and medium (n = 3) categories. Digital firms were chosen due to an interest in studying the assumption that they have been relatively less affected by the COVID‐19 crisis, given their existing technological focus (McKinsey, 2020). Table 1 provides an overview of the cases.

**Table 1:** Overview of the case firms

		**Firm size**				
**Firm**	**Product/service**	Turnover (INR‐Crore)	Employees	**Founded**	**TMT type (number of TMT members)**	**Size classification**
Case 1	Enterprise resource planning (ERP)	4.00	16	2017	Transnational TMT	Micro
					3 TMT‐EDs	
					2 Family directors and 1 ED	
Case 2	Software, infrastructure services and artificial intelligence (AI)	1.9	25	2013	Transnational TMT	Micro
					3 TMT‐EDs and 3 Founders	
Case 3	Technology solutions	0.6	10	2017	Domestic TMT	Micro
					3 TMT‐EDs	
					2 Founders and an ED	
Case 4	Internet of Things (IoT)	15.00	56	2015	Domestic TMT	Small
					5 Founder TMT‐EDs	
					1 Transnational advisory director (TMT‐NED)	
Case 5	Software services	19.00	62	2014	Domestic TMT	Small
					4 Executive directors	
					Domestic	
Case 6	Security services	198.00	350	2000	Transnational TMT	Medium
					4 TMT‐EDs and 2 TMT‐NEDs	
Case 7	Cyber security	180.00	252	2009	Transnational TMT	Medium
					4 TMT‐EDs and TMT‐NED	
Case 8	AI/digital media	230.00	450	2002	Transnational TMT	Medium
					3 TMT‐EDs and 3 TMT‐NEDs	

To ensure deeper contextualization, we captured contextual explanatory reasoning as best as possible within the time constraints. In order to better understand the firm and Indian digital industry context, we interviewed multiple stakeholders. These comprised (a) strategic business leaders (directors or CEOs) who were involved in developing strategies to deal with the COVID‐19 challenges and who had knowledge of the organizational architecture. It should be noted that when we discuss Indian SMEs, TMT terms – including ‘director’ and ‘CEO’ – as well as closely related TMT member titles are used interchangeably (e.g. Puthusserry *et al*., 2021).^3^ (b) Where a company had a formal board, we selected executive (CEOs) and non‐executive directors based in India and, if relevant, their foreign branches. (c) We also interviewed industry experts who are knowledgeable on the trends within the sector to more fully understand and contextualize the influence of COVID‐19 for these firms.

Two phases of interviews were conducted with respondents. Phase 1 took place at the end of March/beginning of April 2020, which was a few weeks after the Indian government had advised companies and employees to WFH due to COVID‐19. Phase 2 took place between June and July 2020 and involved follow‐up interviews to further investigate internal and external contingencies and firms’ response strategies. Interviews in both phases lasted between 60 and 90 minutes and covered questions pertaining to the role of board/TMT within organizations, key organizational activities, structure, coordination, the influence of crisis and response strategies. Interviews were supplemented with archival sources to confirm information related to board/TMT, organizational characteristics and strategic approaches gathered in the semi‐structured interviews. Appendix A presents the data sources.

### Data analysis

Due to seeking a contextual explanation, elements of an abductive approach were utilized during our data analysis. An abductive approach acknowledges a relationship between the theoretical and empirical realms of research (Eriksson and Engstrom, 2021). Indeed, Plakoyiannaki and Budhwar (2021: 4) identify that ‘expectations are shaped by preconceptions, worldviews and beliefs rooted in researchers’ experiences and exposure to theories’. Abduction also lends itself to the application of multiple theoretical lenses in order to facilitate iterations between empirical data and theory (Dubois and Gadde, 2002, 2014) in seeking explanatory reasoning for particular phenomena. This process will now be explored.

**Figure 1:**
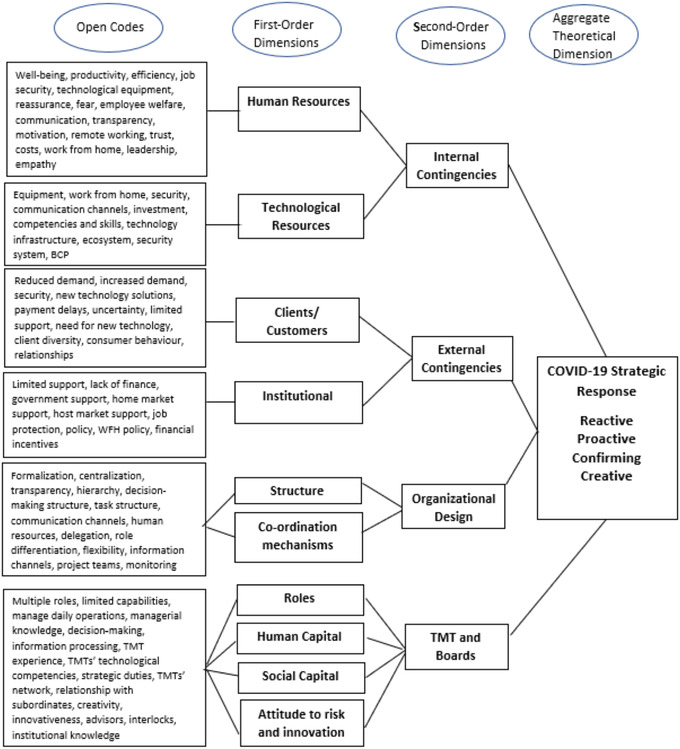
Data structure [Colour figure can be viewed at wileyonlinelibrary.com]

First, the data was analysed by openly coding anything which helped to explain firms’ strategic response to COVID‐19. Taking inspiration from the abductive approach suggested by Dubois and Gadde (2002), we iteratively open coded and followed a ‘matching’ process, where our codes were interpreted in light of theoretical constructs (organizational design, internal and external contingencies and TMT) in order to systematically combine the contextual reality of emerging market SMEs’ responses during COVID‐19 with existing theory to aid explanatory reasoning for decisions and actions. Open codes were combined to form first‐order dimensions and, in turn, these were grouped into second‐order dimensions relating to TMT, organizational design and contingency factors. These stages of data analysis were followed for each case study in isolation by two members of the research team; one who had direct expertise of the Indian context and another who did not have any prior contextual knowledge but had expertise in the applied theories. This helped ensure objectivity and balance between empirical coding and theory matching (Kovács and Spen, 2005). Once this was complete, both researchers met to synthesize the codes and dimensions, compare findings across cases and interpret findings again in light of further existing theory (contingency view of organizational design and UET). This resulted in the identification of a typology of four differentiated strategic responses to COVID‐19. Figure 1 presents an overview of our data structure.

Data quality and reliability was ensured by a clear and transparent data collection and data analysis strategy (Yin, 2018). Appendix B presents an overview of the cases, TMT and organizational design elements with indicative quotes. The use of interviews and archival data facilitated triangulation (Gill, Gill and Roulet, 2018; Lincoln and Guba, 1985), which ensured that all dimensions of the phenomenon had been studied and consequently enhanced the accuracy and validity of contextualized explanations (Dubois and Gadde, 2002). Participants were also invited to ‘member check’ transcripts to confirm the interpretation of key information, and our use of archival sources to cross‐check interview data helped to determine construct validity. Furthermore, as suggested by Yin (2018), matching of empirical and theoretical constructs helped develop correspondence between a theoretical or conceptual pattern and an observed or measured pattern, enhancing internal validity.

## Discussion of findings

The findings revealed commonalities and differences between the firms’ organizational design dynamics such as structure, coordination mechanisms and leadership team (i.e. TMT and board). These organizational factors did appear to form patterns relating to the case firms’ strategic actions and behaviours towards COVID‐19. To fully understand the case firms, we followed Damodaran's (2015) lifecycle stages to classify the firms into start‐ups (Cases 1 and 3), young growth (Cases 2, 4 and 5), advanced growth (Cases 6 and 7) and mature growth (Case 8), which revealed further patterns relating to the organizations’ response strategies.

The findings will start with a discussion of the external and internal contingencies created by the COVID‐19 pandemic. This will be followed by an analysis of the case firms’ COVID‐19 response strategies. Table 2 presents a summary of the findings.

**Table 2:** Summary of key findings

**Lifecycle phase**	**Contingencies**	**TMT**	**Organization design**	**Strategic response**	**Growth strategy**
**Start‐up** Micro firms	**External** Lack of spread in customer base Institution void in the home market	**TMT‐EDs** Strategic and operational roles Homogeneous HC and SC Limited industry‐specific experience and connections – Limited ability to address external contingencies – Need to expand capabilities and knowledge to be innovative	Informal and centralized founder‐centric hierarchical structure, decision‐making authority, coordination and control mechanisms – Limited ability to come up with transformational strategies to deal with internal contingencies	**Reactive** Focused on internal adaptations Affected by lack of breadth and limited depth of board or TMT capital and amorphous design	***Inertia*** Retrenchment approach to saving cost – Achieved through redundancy and outsourcing of technology development activities
**Young‐growth phase** Micro and small firms	**External and internal** Lack of diversity in customer base Institution void in the home market	**TMT‐EDSAdvisor‐NEDs** Heterogeneous HC and SC – Diverse expertise, experience and external and internal network – Ability to innovate – Risk‐taking and innovative approaches	Early phase of differentiating TMT and functional management team Formalization – Streamlining tasks, clarifying roles, responsibilities – Direct, centralized coordination, controlling and monitoring Flexible organizational design	**Proactive** Focused on both external and internal contingencies Facilitated by diverse board‐level human and social capital Developing capabilities through reconfiguration	**Market development** Retrenchment to increase efficiency and performance Redundancy and streamlining business activities. Outsourcing supporting functions
**Advanced‐growth phase** Medium‐sized firms	**External and internal** Diversity in client base Emphasis on employee welfare and well‐being Focused on core technological domains	**TMT‐EDs and TMT‐NEDs** Clear role specification between TMT and senior managers – TMT‐ NEDs strategic role – TMT‐EDs operational and functional role Depth of TMT level human and social capital facilitated – Experiential learning – A greater understanding of industry dynamics – Ability to adapt the organizational design	Decentralized and delegated formal coordination, monitoring and governance Remobilizing of human resources Restructuring and reprioritizing tasks and activities Transparency in communication IT system‐facilitated communication and monitoring	**Conforming (stable market position)** Internal and external contingencies – External contingencies relevant to immediate stakeholders Facilitated by the depth of TMT‐level social and human capital and technology	**Market penetration** Additional investments in human and technological resources to enhance employee welfare and customer relationships
**Mature‐growth phase** Medium‐sized firms	**External and internal** Clients in multiple technology domains – Flexible with customers – Learning from customers Human and technological resources	**TMT‐EDs and TMT‐NEDs** Heterogeneous social and human capital – Provide strategic flexibility to control and configure organizational design	Diverse tasks structure Formal, delegated and decentralized organizational structures and coordination mechanisms Multiple information channels – Helped them integrate, build and reconfigure internal and external competencies	**Innovative (established market position)** Developing new product/solution Facilitated by diversity in TMT human and social capital and having formal and established organizational designs	**Product development and diversification** Innovation of new products for existing customers and new markets

### COVID‐19‐related contingencies influencing firms

As seen in Table 2, the case firms identified that COVID‐19 presented several external and internal contingencies. The emphasis the interviewees gave to each of these contingencies varied by the organizational lifecycle. The start‐up (Cases 1 and 3) and young‐growth (Cases 2, 4 and 5) firms had a greater focus on addressing external contingencies due to their restricted external knowledge capabilities and resources to sense challenges and identify opportunities. In contrast, the advanced (Cases 6 and 7) and mature (case 8) growth firms put emphasis on both external and internal contingencies due to their extensive client base and systematic and formalized organizational designs.

### External contingencies

#### Clients/customers

All respondents identified that COVID‐19 did, in some ways, create opportunities for digital companies through work being able to be conducted remotely, and ability to access increased clients ‐ especially for firms who were not previously utilizing technology, now needing to implement technology solutions. However, the start‐up and young‐growth firms included in this study reported that COVID‐19 had a huge disruption for their customer base, which created a cumulative effect on their own operations. The director of a start‐up that provides enterprise resource planning (ERP) service solutions to airline, travel and tourism companies (Case 1) indicated:

We thought we would be fine as everything is going digital, but we feel the impact now… it impacted our customers. One of our projects is called off …. We don't know what will happen. We have delays in payments too… some contract renewals are also got delayed. We are not sure if they will renew the contract. (Director, Case 1)

When the start‐up firms (Cases 1 and 3) were asked what they will need to change to remain sustainable during and post‐COVID‐19, the interviewees appeared to be unsure and identified that their smallness and newness meant that they need to expand their current capabilities and knowledge to open up new markets. Indeed, both of these start‐up firms had small homogeneous TMTs. Their TMTs were found to occupy multiple roles in their organizations, including the daily responsibility for managing operations, which they identified was limiting their time and ability to expand the business into innovative emerging areas such as artificial intelligence (AI), machine learning (ML), robotics and the Internet of Things (IoT).

The young‐growth firms (Cases 2, 4 and 5) identified that their TMTs’ diverse experience and expertise were crucial in aiding their ability to innovate, which was helping them mitigate the issues they were facing in relation to a reduction in client orders in the short term. In comparison to the start‐up cases, they exhibited a heterogeneous TMT comprised of executive directors with diverse human and social capital. It also emerged that these firms had experienced industry experts as advisory directors. The young‐growth firms were in the early phase of differentiating their board of directors from their management team through delegating functional level responsibilities. Specifically, it was identified that directors’ connections facilitated learning, capability development and the development of new business contacts (Puthusserry *et al*., 2020). This opened up opportunities for new clients who needed digital services due to COVID‐19. Young‐growth firms had to rapidly develop new digital capabilities to enhance their offerings and enable entry into new markets. The founder CEO of Case 4 illustrates this:

Our advisor in the USA had connections with the owner of a shipping company. He realized that we would be able to help develop digital freight movement monitoring mechanisms for them so he suggested for us to prepare the solution… He provided us with support as he has prior experience and connections in the shipping industry. He saw opportunities there. It was because of his assurance that we decided to diversify into shipping during the COVID period. (Founder CEO, Case 4)

The investigated advanced‐growth firms (Cases 6 and 7) highlighted that their diverse client bases helped them mitigate the influence COVID‐19 had on them. They also identified that COVID‐19 had caused a shift in consumers’ behaviour to adopt more technology, which was presenting opportunities in their existing technology domain. Their ability to rapidly capitalize upon the new markets was thought to be related to their delegated organizational structure, with clear role differentiation between their board of directors and TMT. Their board included both executive and non‐executive directors who dealt with long‐term strategic issues and other fiduciary duties. In contrast, the TMT included functional and divisional heads, and their responsibilities were mainly related to operational issues. Indeed, a non‐executive director identified that COVID‐19 allowed them to follow a market penetration strategy. This is illustrated by the founder‐director of Case 7:

We are a completely cloud‐based digital company. Cloud adoption was a problem in India, but the current scenario made everybody revisit their cloud adoption strategies. That had a positive fallout for us. Many companies are moving to the cloud now and willingly adapting to the cloud platform. Many of the new companies are coming to our cloud services. (Founder‐Director, Case 7)

Respondents from the mature‐growth firms (Case 8) also identified that working closely and being flexible with customers afforded opportunities to develop institutional knowledge and innovate to meet rapidly changing market demands. This is consistent with prior literature, which argues that customers are not only important for sales but can also represent key resources for organizational learning and innovation (Fjeldstad and Sasson, 2010; Idris, Saridakis and Khan, 2021).

### Institutional‐related bottlenecks

The second theme of institutions identified the challenges an emerging market presents during a crisis such as COVID‐19, influencing the case firms’ ability to respond. TMTs of the start‐ups and young‐growth firms, who all specialize as technology delivery centres to counterparts in developed markets, indicated that the lack of financial packages from the Indian government had compelled them to scale down and streamline their operations. For example, the CEO of Case 4 said:

We had to layoff around 20% of the employees as we are on 50% capacity now. It is the only way to reduce our operating cost. We would have avoided it if there was any financial support from the state or central governments. The state government has instructed us not to make any redundancies now but we realized that keeping poor performers is not viable. We also wanted to increase the efficiency. (CEO, Case 4)

However, the implications of lack of home market institutional support varied greatly between start‐ups and young‐growth sample firms, where this actually enacted a proactive response amongst young‐growth firms. This is demonstrated by the CEO of Case 2:

Unlike the USA or UK, where the government provides financial support in the form of interest‐free loans, furloughing employees, we did not have any support here. Initially, we were confused as we never faced similar situations, but we consulted with people, including those who faced the 2008 financial crisis. We realized that we wouldn't survive if we didn't act quickly. We used our network to learn about the situation and get a new business. (CEO, Case 2)

Conversely, the response of a director of a start‐up technology solution company investigated in the Indian context insinuated that a relative lack of institutional support received in their host market undermined their ability to be proactive:

We got financial support from the [UK] government in the form of interest‐free loans or other incentives. It is a huge relief. That will help me run for 1 year. However, the situation in India is different. The majority of our employees are in India. We are only five people in the UK. We will have to lay off people in India if the situation worsens. Redundancy is a huge risk as it will be difficult to rebuild in the post‐COVID situation. (CEO, Case 1)

The responses of the industry experts also implied that they believed start‐ups and young‐growth firms in the Indian context, with high liabilities of newness and smallness, would not survive without financial incentives from government and institutional agencies. Respondents stated that it is important to protect jobs in the world's largest IT and ITeS sourcing destination (NASSCOM, 2017):

Government support is really important to protect employment, not just for companies but also for the Indian IT sector… It is difficult for small and highly specialized firms but those who have specialisms in multiple knowledge domains such as AI and ML are able to respond quickly. They will have to identify the opportunities and adapt quickly. They will not survive if they can't. (Industry Expert 1)

### Internal contingencies

Responses revealed that COVID‐19 presented unique internal contingencies that differ from other crisis events any of the organizations had faced or could have anticipated. These internal contingencies were intertwined with their ability to respond to external contingencies.

### Human resources

All respondents identified that COVID‐19 presented unique circumstances relating to how they configure and manage their employees. Remobilizing human and other resources, which are required to conduct work (e.g. technology), was most of the firms’ first major strategic challenge of the crisis. This required restructuring and reprioritizing tasks and activities to accommodate WFH. In the case of the mature‐growth firms, they had well‐established and formalized organizational structures and systems, which they identified to have designed to help with managing uncertainty and change. However, they did identify that the ongoing challenges they faced relating to human resources as a result of COVID‐19 are not something they believed they could ever have planned for. Despite this, their formal but delegated and decentralized structure and multiple information channels provided flexibility and coordinated their complex task structure, helping them respond with relative agility and human‐centric skills. They also highlighted that their TMTs’ human and social capital was important in helping communication processes to reassure staff of job security. Case 8 identifies:

Our CEO is a philanthropist. He meets employees every week virtually. In all these meetings, one thing he emphasizes is that take care of your family, your community and come back and work. We are very flexible with time now. He also emphasized that nobody will be laid off… We now have much more frequent communication and direct involvement in the decision making. It is not only important for boosting their morale but for the efficient management of tasks. (Media Director, Case 8)

Employee welfare and well‐being was identified to be a major internal contingency for advanced‐growth firms. For example, the senior executive of Case 7 identified that:

We have to be a little bit more sensitive in this situation. The prolonged period of working from home is also not a very conducive situation, especially when the distressing things happening around can make people a bit traumatized. I touch base with my team every day. Earlier it was once in a month. Being sensitive is very important. (Senior Executive, Case 7)

Respondents from advanced‐growth firms noted that adopting a transparent approach and engaging in open dialogue regarding future plans and strategies of the company was vital in fostering employee motivation, which consequently aided their company to keep functioning. They also noted that the new distributed remote independent WFH working model has led to direct and centralized monitoring and governance mechanisms. For example, the senior executive of Case 7 also mentioned:

We now have a more streamlined and centralized governance mechanism. Our directors are directly heading each functional team. For example, I am the sales head. I have been directly involved in all sales‐related decisions. I interact with clients and our teams. It is important to get their trust. (Senior Executive, Case 7)

In contrast, start‐ups and young‐growth firms did not highlight employee welfare and well‐being as a major contingency. Instead, they highlighted that the external challenges had forced them to streamline the business activities and adopt a retrenchment strategy. However, responses also varied between start‐ups and young‐growth firms; young‐growth firms adopted full retrenchment strategies to enhance efficiencies and performance, while start‐ups focused on reducing costs through outsourcing of software development.

### Technological resources

Despite being digital firms, respondents from all four categories of firms investigated in the Indian context emphasized developing technological resources and capabilities to address the internal contingency created by WFH policies. Start‐ups’ and young‐growth firms’ simple and informal task structures and direct coordination mechanisms appeared to be advantageous in helping them adapt their operations and policies to facilitate WFH. However, responses inferred that developing technological infrastructure and capabilities to address the internal contingency created by WFH policies was more of a major challenge for advanced and mature‐growth firms – perhaps owing to their more complex, delegated organizational task structures and formal coordination and decision governance mechanisms. While these firms reported initial difficulties moving from a highly integrated office‐based working model to a distributed remote independent working model, they also highlighted that having a technology ecosystem enabled endpoint control and a more distributed working model. This is illustrated by Case 6:

Security is a sensitive domain. Customers are particular about where the work is being conducted. The facilities, the locations, the access controls, segregation of duties, the various maker check controls and all compliance and monitoring mechanisms, governance mechanisms, everything is important for the customer – all changes when you go into remote working. (Founder‐Director, Case 6)

Business continuity plans and practices were found to provide advanced and mature‐growth firms with the structure and templates necessary to respond quickly and successfully to the COVID‐19 shock. This is illustrated by the director of Case 8:

We play different scenario games like how to deal with the situation when 50% of our employees are not able to work or decided to leave the company. There is a template of all such scenarios. (Director, Case 8)

The directors of both advanced and mature‐growth firms investigated in this study also reported that they had to make additional investments in developing their technological infrastructure and ecosystem due to the unexpected nature and scale of the COVID‐19 crisis.

### A typology of emerging market SMEs’ COVID‐19 response strategies

**Figure 2:**
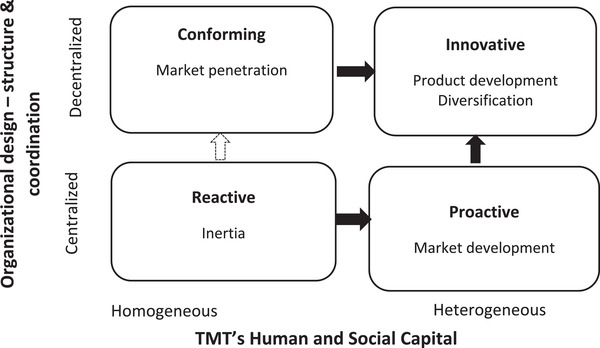
A typology of Indian digital SMEs’ COVID‐19 response strategies

Across the cases, four dominant response strategies emerged: *reactive*, *proactive*, *conforming* and *innovative*. These response strategies unexpectedly aligned with the lifecycle stage of the case firms, which signals the important role that organizational context plays when considering crisis response strategies. Figure 2 presents a typology of the response strategies, and a summary of the characteristics of each response strategy is provided in Table 2. Each response strategy will now be discussed.

It was found that a *reactive* strategy response was followed by start‐up case firms (Cases 1 and 3) in order to mitigate their organizational contextual factors, which presents differentiated external and internal contingencies created by COVID‐19 compared to firms of other types. Consistent with prior literature, these firms possessed an informal and centralized hierarchical structure, decision‐making authority, coordination and control mechanisms (De Massis, Eddleston and Rovelli, 2020; Grimpe, Murmann and Sofka, 2019; Ling *et al*., 2008). Their TMT context is characterized by homogeneous TMT‐level human and social capital (Haynes and Hillman, 2010), which limited their ability to come up with transformational strategies to deal with the crisis. These characteristics were found to help them address the internal contingencies related to human and technological resources through a retrenchment approach and centralized autonomous controlling. However, their lack of heterogeneity in their board capital (breadth) and limited experience and understanding of the sector (depth) affected their cognitive diversity and ability to identify appropriate opportunities and make a rational decision to address external client‐specific contingencies (Adner and Helfat, 2003). Hence, we argue that TMTs’ bounded rationality and cognitive limitations influence a firm's decision to focus on internal contingencies, which may further limit their ability to develop positive stakeholder relationships (Bundy *et al*., 2017). This is consistent with Storey and Skyes’ (1996) and Lai *et al*.’s (2016) observations that small firms with less formalized and amorphous structures and limited TMT human capital may be less resilient to market shocks. Furthermore, Merendino and Sarens (2020) identify the importance of managerial cognition as a determinant of crisis response. Since micro and start‐up firms are mainly run by their founders, consistent with Zuzul and Tripsas (2020), we argue that founders’ background, future vision, confidence, choice of market, over‐reliance on existing customers and market are the sources of their inertia.

The findings revealed that firms who had *proactive* responses to COVID‐19 were Indian young‐growth firms (Cases 2, 4 and 5). These firms were found to benefit from the diversity in their TMT context (heterogeneity in TMT‐level human and social capital) (Haynes and Hillman, 2010; Keck, 1997), yet they lack well‐developed organizational designs, formalization of roles, tasks, communication, decision‐making and coordination mechanisms (organizational context) (Burton *et al*., 2019; De Massis, Eddleston and Rovelli, 2020). Concurring with prior research by Dowell, Shackell and Stuart (2011) and Merendino and Sarens (2020), it could be suggested that their heterogeneity or breadth of TMT‐level human and social capital provided the flexibility not only to adapt the organizational design but also to engage in more risk‐taking and innovative approaches to address external client‐specific contingencies. This is reflected in their response of proactively entering a new market (market development) (Beckman and Burton, 2008; Colombo and Grilli, 2005; Lahiri *et al*., 2019). External contingency related to institutional voids in their home market (external environmental context) was also suggested to affect proactive behaviour (Doh, Tashman and Benischke, 2019; Khanna and Palepu, 1997). TMTs of these firms appeared to be making conscious efforts to formalize organizational designs through the retrenchment approach of outsourcing key non‐technical support functions, the streamlining of tasks and the clarification of roles, responsibilities and lines of reporting, to prepare for future COVID‐19 shocks (Grimpe, Murmann and Sofka, 2019). This is consistent with observations that directors and/or CEOs of small firms centralize decision‐making authority to mitigate coordination, communication and/or formalization challenges (De Massis, Eddleston and Rovelli, 2020; Ling *et al*., 2008). Furthermore, the retrenchment approach helps TMTs further develop their core competencies and social capital with diverse external stakeholders (Bundy *et al*., 2017).

A *conforming* strategic response strategy was followed by the advanced‐growth firms (Cases 6 and 7). These are medium‐sized firms, and their organizational context is characterized by a decentralized, delegated and non‐hierarchical task structure with formal coordination, monitoring and control systems (Burton *et al*., 2019; Haeussler, Hennicke and Mueller, 2019). The TMT context, TMT‐level human and social capital of these firms had significant depth but lacked breadth (Haynes and Hillman, 2010). More specifically, the directors of these firms were mainly technically qualified people with experience and expertise in their core knowledge domain (Colombo and Grilli, 2005) and had interlocking directorship and embeddedness in their focal industry (Haynes and Hillman, 2010). Industry‐specific experience and personal trust‐based relationships were found to not only facilitate directors’ experiential learning but enhance their understanding of industry dynamics (Puthusserry *et al*., 2021) and helped adapt the organizational design to address both internal and external contingencies. The executives reported that their experience and connection within the organization and the introduction of new technology facilitated them to adopt delegated decentralized monitoring and governance mechanisms to address the internal contingencies created by WFH policy. TMTs’ human (experience) and social capital (connections) within the organization and employees’ prior experience of working together and repeated interactions (Srikanth and Puranam, 2014) facilitated asynchronous tacit coordination mechanisms (George, Lakhani and Puranam, 2020) of decentralized interdependent task structure. The technology provided a system for remote monitoring of actions across firms (Kotha and Srikanth, 2013; Srikanth and Puranam, 2014). Depth of TMT‐level capital and their decentralized and formal organizational design facilitated a *market penetration strategy* of penetrating further into their existing markets.

Finally, an *innovative* response strategy was followed by mature‐growth case firms. The TMT context of these firms is characterized by heterogeneous TMT‐level human and social capital (Haynes and Hillman, 2010; Zorn *et al*., 2017), and their organizational context (i.e. organizational design) consists of diverse tasks structure, formal, delegated and decentralized organizational structures and coordination mechanisms (Burton, Obel and Håkonsson, 2020). Heterogeneity in TMT‐level human and social capital provided them with the strategic flexibility to control and configure their organizational design (De Villiers, Naiker and Van Staden, 2011; Keck, 1997) and helped them ‘integrate, build and reconfigure internal and external competencies’ (Teece, Pisano and Shuen, 1997: 516) to address COVID‐19‐related challenges and opportunities. This is reflected in their innovative approach of developing new products/solutions to mitigate their own challenges (product development) as well as assisting other stakeholders (diversification) in navigating the evolving COVID‐19 pandemic successfully (Balachandran, Wennberg and Uman, 2019). Diversity in TMT human and social capital and having formal and decentralized organizational designs, such as task structure, formal role specification, decision process and information channel, were essential in facilitating a *product development strategy*.

### Contributions and conclusions

Research to date identifies the importance of organizational design and the role boards and TMTs have in influencing crisis response strategies, yet these literatures have evolved separately with limited cross‐fertilization (De Massis, Eddleston and Rovelli, 2020; Lewin and Stephens, 1994). In this study, we amalgamated and explicated their utility in order to study (1) How do internal and external contingencies of COVID‐19 influence emerging market SMEs? and (2) How do organizational design and board capital shape the strategic actions taken to overcome COVID‐19 contingencies?

In addressing research question 1, the findings identified that the external contingencies associated with COVID‐19 were influenced by the digital industry context (clients/customers) and the national environmental context (institutions). Furthermore, the existence and prevalence of these challenges were dependent on the lifecycle stage of the SME (i.e. organizational context). We identified that internal contingencies were associated with human resources and technological resources, which are reflective of the inherent resource constraints SME's may face during a crisis. However, the extent to which firms experienced internal contingencies was dependent upon their lifecycle stage.

Addressing research question 2, we provide new knowledge, which responds to calls for research that analyses how longstanding rules and theories translate in new crisis contexts (Foss, 2020; Wenzel, Stanske and Lieberman, 2020). We illustrate the combinative effect organizational (organizational design – structure and coordination mechanisms) and TMT context (TMT roles, human capital, social capital and attitude to risk and innovation) can have in shaping the strategic actions taken by firms to overcome COVID‐19‐related contingencies. In Table 2, we illustrate how different levels of TMT‐level capital, combined with specific organizational design elements, helped to provide explanatory reasoning for why Indian digital SMEs had four dominant response strategies during the early stages of COVID‐19 (*reactive*, *proactive*, *conforming* and *innovative*; see Figure 2). Our findings illustrate that the response strategy followed appeared to align with their organizational context (i.e. lifecycle) and was impacted by both the industry and environmental context (i.e. India) (see Table 2). Based on these findings, we make a number of contributions.

First, we extend the crisis management literature through illustrating that firms' COVID‐19 strategic response should seek fit and alignment with environmental, organizational and TMT contextual characteristics. Our findings reinforce the need for a contingency approach (Galbraith, 1973; Mintzberg, 1979) – where there is no ‘one size fits all’ crisis strategic response (Foss, 2020; Wenzel, Stanske and Lieberman, 2020). However, we illustrate that strategic response patterns emerge for firms who have similar organizational designs and levels of board/TMT capital. Related to this, through our typology of strategic responses, we expand prior literature on the role of organizational design and TMTs during crises. Our typology (Figure 2) and findings (Table 2) provide much‐needed new insights into how internal organizing practices and resources are associated with strategic responses by firms experiencing prolonged crises (Bundy *et al*., 2017; Wenzel, Stanske and Lieberman, 2020; Williams *et al*., 2017). From the findings, it could be suggested that fast and original crisis responses should balance both planned and structured activities (reliant upon organizational design) with impromptu entrepreneurial actions (influenced by TMT/board capital) – such combinatory approaches are crucial for firms based on emerging markets to effectively respond to prolonged external shocks.

Our findings further extend the crisis management literature by illustrating how SMEs can enact growth strategies during a crisis. The findings identify that mature‐growth firms engaged in new product development to either establish their position in existing markets (product development) and/or to enter new markets (diversification). To achieve this, an established decentralized structure with formal integration and monitoring system and diverse TMT‐level human and social capital was required. In other words, board/TMT capital and organizational capabilities complemented each other to facilitate superior performance for the Indian digital SME firms included in this study. In contrast, start‐ups, which had an informal design and limited TMT‐level social and human capital, exhibited inertia in response to COVID‐19. Start‐ups are often run by the founder, and consistent with De Massis, Eddleston and Rovelli (2020) and Zuzul and Tripsas (2020), our findings identified that sources of inertia within our case firms arose from the founders’ background, future vision, confidence, choice of market, over‐reliance on existing customers and market.

Our research also extends research on TMTs and, in particular, responds to calls for research on how TMTs and their corresponding social and human capital can support firms’ crisis responses (Merendino and Sarens, 2020; Verbeke and Yuan, 2020). In doing so, we extend existing UET literature and provide micro‐level firm insights into TMT and board diversity during a crisis (Merendino and Sarens, 2020; Mumford *et al*., 2007; Verbeke and Yuan, 2020). Concurring with prior research (Crossland *et al*., 2014; Keck, 1997), our findings illustrate how heterogeneity or breadth of TMTs’ social and human capital provided the flexibility to not only adapt organizational design but also engage in a more risk‐taking and innovative approach to address the external and internal contingencies created by COVID‐19. This is reflected in the ‘proactive’ market development and ‘innovative’ product development responses of young‐growth and mature‐growth case firms, respectively. Contrary to this, the depth of board/TMT capital (Colombo and Grilli, 2005) facilitated the advanced‐growth case firms’ market penetration strategy of penetrating further into their existing markets, which helped address the initial crisis shock. Although homogeneity in board capital enabled the survival of start‐ups, their lack of breadth and depth of board or TMT capital led to bounded rationality (Cohen, Bingham and Hallen, 2019) and emotional responses (Merendino and Sarens, 2020). It affected their ability to make an optimal response to deal with the crisis due to their limited cognitive diversity (Classen *et al*., 2012).

Moreover, our study contributes to calls for research by De Massis, Eddleston and Rovelli (2020) and De Massis and Rondi (2020) on how leadership in small firms shape organizational designs to implement a strategy to address crisis contingencies. The findings revealed that differences in TMT configurations of start‐up and scaling‐up firms influenced their choice of organizational design and strategic response. The CEO‐centric TMT structure of start‐ups limited their opportunity exploitation. This was suggested to then have influenced their reactive approach and inertia towards the response to the COVID‐19 crisis. In contrast, participative TMTs in start‐up firms enabled their proactive response and market development growth strategy (De Massis, Eddleston and Rovelli, 2020). The findings suggested that organizational design plays a lesser role in crisis response strategies of start‐up and young‐growth case firms compared to our sample of advanced‐growth and mature‐growth firms. Indeed, smaller firms with fragmented and centralized organizational design – which are typical of start‐ups and young‐growth firms – are said to over‐rely on managerial judgement due to limited resources, which can be inaccurate and clouded with emotional reactions during a crisis (Eduardsen and Marinova, 2020), leading to internal coordination challenges and poor decisions. Our findings reinforced Storey and Skyes’ (1996) and Lai *et al*.’s (2016) observations that small firms with less formalized and more fragmented structures and with limited human resources may be less resilient to market shocks.

We also expand prior literature on organization design by providing much‐needed empirical insights into the role it can have in influencing crisis response strategies (Foss, 2020; George, Lakhani and Puranam, 2020; Williams *et al*., 2017). Whilst prior research suggests that organizational design may influence the strategic flexibility and innovation of firms, research to date is inconclusive and largely based on theoretical assumptions (Carmeli and Schaubroeck, 2008; Lai *et al*., 2016). Since we highlight types of organizational design that appear more closely associated with greater resilience to black swan crises (Foss, 2020; George, Lakhani and Puranam, 2020), our findings have direct relevance for practitioners and policymakers in designing effective policy to navigate future black swan crises. This is further germane given that while COVID‐19 is a unique crisis, unfortunately, black swan events have been predicted to become more common (Taleb, 2007).

We further refine organizational design research (see De Massis, Eddleston and Rovelli, 2020; Ennen and Richter, 2010) and contribute to the contingency perspective by providing new insights illustrating the interdependencies of organizational design elements (leadership, structure and coordination) simultaneously in order to understand how our case study digital SMEs respond during the COVID‐19 crisis. Our findings reveal that medium‐sized Indian sample firms (advanced and mature‐growth firms) with delegated and systematic organizational design and practice presented unique external and internal contingencies associated with the crisis. This is consistent with Clement and Puranam's (2018) and Puranam, Alexy and Reitzig's (2014) observations that medium‐sized firms’ organizational design had developed over time as a result of TMT‐level social and human capital, learning and competency development. Furthermore, we illustrate how medium‐sized firms’ decentralized and systematic organizational design provided fluidity and facilitated reconfiguration of resources and capabilities during the crisis (Schreyögg and Sydow, 2010). However, TMT‐level human and social capital differences seemed to have influenced the strategic responses of advanced and mature‐growth case firms. For example, in advanced‐growth case firms, the depth of TMT‐level human (experience) and social capital (connections) within the organization and industry and technology infrastructure facilitated asynchronous tacit coordination and monitoring of decentralized interdependent task structures (George, Lakhani and Puranam, 2020; Kotha and Srikanth, 2013; Srikanth and Puranam, 2014). It also facilitated their market penetration strategy. The findings identified how homogeneous TMT capital and routinized organizational design practice (Nicolini, 2012) influenced advanced‐growth case firms’ resilience to change, which in turn reflected their ‘conforming response’ to COVID‐19 (Seidl and Whittington, 2020). In contrast, mature‐growth case firms’ TMTs’ social and human capital heterogeneity, combined with their delegated organizational design, helped them develop a balanced internal and external crisis mitigation approach through innovative product development and diversification strategies (Bundy *et al*., 2017).

Our findings also provide new knowledge, which contributes to Ahlstrom *et al*.’s (2021) call for research to understand whether different types of TMT‐level human and social capital may affect organizational resilience to crises. Our findings show that diverse TMT‐level capital, task structures and reporting mechanisms created challenges for medium‐sized firms’ internal coordination and ability to adapt to external contingencies promptly. However, new information and communication technology facilitated the coordination and monitoring of task structure.

Crises often result in widening the equality gap in emerging markets (Cowling, Liu and Zhang, 2020), consequently, our research extends prior research on the emerging market SME context (Amankwah‐Amoah, Khan and Wood, 2021; Miller *et al*., 2021) and the influence crises have for them (Bundy *et al*., 2017). Our findings identify that the limited support offered by the Indian government impacted the SMEs. This signals that environmental and institutional contexts will influence the magnitude of the impact of COVID‐19. However, we found that the lack of government support was more prevalent for the Indian start‐up case firms, who also had limited TMT and board capital. Therefore, we identify that start‐up firms’ TMTs and organizational context, combined with a lack of external institutional support and internal knowledge, will negatively influence the capabilities and resources they need to engage in strategic actions focused on renewal or innovation (Covin and Slevin, 1989; Shirokova *et al*., 2020).

Furthermore, our findings reinforce prior literature on the role of organizational and TMT context (Autio *et al*., 2014; Goshal and Bartlett, 1994; Shepherd and Rudd, 2014). Micro and small firms, characterized by centralized and fragmented organizational structure, coordination and management practices (Burton *et al*., 2019), only emphasized external clients and institution‐related contingencies that influenced operations and performance. The findings suggest that centralized decision autonomy which was evident in small Indian firms included in this study, helped to mitigate coordination, communication and/or formalization challenges (De Massis, Eddleston and Rovelli, 2020). Whilst these firms possessed flexible and amorphous structures, which allowed them to respond quickly, their limited external knowledge capabilities and resources played a stronger role in influencing their strategies. This finding is consistent with the notion that new ventures or small firms’ competencies and performance are mainly influenced by the broader functional experience (Beckman and Burton, 2008) and technological competencies (Haeussler, Hennicke and Mueller, 2019) of founders/entrepreneurs.

### Practical and policy implications

Overall, our findings have important practical implications illustrating to managers that not all SMEs are vulnerable during crises (Lai *et al*., 2016). We demonstrate through Figure 2 and Table 2 how firms within emerging markets can drive performance through market penetration, product development and diversification growth strategies during crises. Consequently, managers can use these lessons to reconfigure their TMTs and boards to ensure TMT/board capital heterogeneity to help mitigate the influence of future shocks.

Furthermore, our findings have important policy implications for an emerging market like India. Figure 2 and Table 2 can be used by policymakers as diagnostic tools to identify the potential influence of a crisis for firms at different lifecycles and with particular board and organizational design characteristics within particular regions. This can then help facilitate proactive interventions in markets, industries or even firms.

### Limitations and future research

Despite the important contributions of our study, it has limitations that offer opportunities for future research. Whilst our discussion and contributions illustrate the possibility of analytical generalization (Yin, 2018), our findings might not be generalizable across other emerging markets or contexts. Therefore, future research could study the applicability of our typology across firms in different markets and contexts. Since our research was cross‐sectional and studied the initial 4 months of COVID‐19, future research could provide a longitudinal analysis of firms’ COVID‐19 strategies to explore how strategies evolve over time and the internal and external enablers of change during the crisis as well as post‐crisis. Finally, the qualitative nature of our study means that although the rich, contextualized insights we identify are important and valuable to aid contextual explanatory reasoning, they are not causal. When more data becomes available in the future, researchers could employ quantitative research approaches – including quasi‐experimental research designs that exploit variations at country/industry or more granular levels. This will help to derive causal insights into the role of TMTs and organizational designs in helping firms navigate COVID‐19 and other future black swan crises.

## Footnotes

1 In this paper, we follow the recommendation of an anonymous reviewer and adopt a broad definition of the TMT given our focus on emerging market small and medium‐sized enterprises (SMEs), which encompasses both senior executive directors as well as wider non‐executive directors of the board. In the discussion of our analyses, and elsewhere, where a particular role traditionally resides more with key executive directors (e.g. CEO) or non‐executive directors, we indicate this by stating either ‘TMT‐ED’ or ‘TMT‐NED’, to refer to predominately executive directors or non‐executive directors, respectively.

2 Based on the Indian Ministry classification of micro, small and medium enterprises (MSMEs) (Government of India, 2020). According to this, micro firms have annual turnover less than INR 5 crore, small firms annual turnover less than INR 50 crore and medium‐sized firms annual turnover less than INR 250 crore.

3 It is important to note that in India, smaller companies often lack formal ‘boards’, and a single high‐ranking TMT member such as the CEO is often considered to represent the ‘board’, where they undertake multiple roles (Puthusserry *et al*., 2021).

## Appendix A

### Data overview

**Table:**

**Firm**	**Respondents**	**TMT units**	**Archival materials**
Case 1	Director of Engineering (India)	TMT‐EDs	Annual report; LinkedIn and Twitter profiles of Case 1 and respondents
	CEO (UK)		
Case 2	CEO/founder (India)	TMT‐EDs	Company website (background); LinkedIn and Twitter profiles of Case 2 and respondents
Case 3	Business director (India)	TMT‐EDs	Annual report; LinkedIn and Twitter profiles of Case 3 and respondents
Case 4	CEO (India) Non‐executive director (USA)	TMT‐EDs and TMT‐NED	Newspaper article on Case 4; LinkedIn and Twitter profiles of Case 4 and respondents
Case 5	Director (India)	TMT‐EDs	LinkedIn and Twitter profiles of Case 5 and respondent
Case 6	2 Founder directors (India)	TMT‐EDs and TMT‐NEDs	Newspaper articles on Case 6 and a TMT‐ED; LinkedIn and Twitter profiles of Case 6 and respondents
Case 7	Senior executive (India) Non‐executive director (Singapore)	TMT‐EDs and TMT‐NEDs	Company website, annual report, press releases from Case 7; LinkedIn and Twitter profiles of Case 7 and respondents
Case 8	Media director (India/USA)	TMT‐EDs and TMT‐NEDs	Company website, online articles on TMT and new products; LinkedIn and Twitter profiles of Case 8 and respondents
Industry expert 1 (IE1)	Director of an Indian software MNE	TMT‐NED	LinkedIn and Twitter profiles of IE1
Industry expert 2 (IE2)	CEO of a technology centre	TMT‐ED	Company website, online articles of IE2, LinkedIn and Twitter profiles of IE1
Industry expert 3 (IE3)	Indian tech journalist	–	Online articles published by IE3
Industry expert 4 (IE4)	Software professional and union leader	–	LinkedIn and Twitter profiles of IE4
Industry expert 5 (IE 5)	AI expert	TMT‐ED	Online articles published by IE5; LinkedIn and Twitter profiles of IE5

## Appendix B

### Summary of leadership and organizational designs of four categories of firms

**Table:**

**Firm type**	**Firm characteristics**	**Leadership**	**Structure**	**Coordination mechanism**
**Start‐up (2 case firms)**	**Average age**: 3 years **Size**: Micro firms Employees (Avg.) = 10 Turnover (Avg.) = $0.3 m **Product/service**: Technology service solution providers	**TMT**: Family directors Average size = 3 Homogeneous TMT‐level HC and SC – Expertise – narrow technology domain – Connections and networks limited to the core domain – Narrow customer segment **Role**: Occupy multiple roles Daily operations responsibility – Client interaction Limited time to develop long‐term strategic decisions Understanding of the internal activities and people Personal relationships with subordinates. Illustrative quote from interviews: *All business comes through me as I deal with clients. When we get a new enquiry, I work with our director of engineering to do a feasibility study. We then work with the team to develop the prototype, and if the clients are happy, we go for commercialization. I am directly involved in all these activities*. (CEO, Case 1)	**Goal**: Achieve cost‐efficiency **Key tasks**: Repetitive and related to their main knowledge domain Illustrative quote from interviews: *We mainly provide technology services and solutions. Our CEO and technical director have expertise in this technology… Customers give us their requirements. We just do what they want. It is usually a routine job*. (Business Director, Case 3) **Structure**:There is no clear organizational structure in terms of the division of labour, role formalization and relationship hierarchy Centralized TMT‐centric, hierarchical organizational architecture	Informal coordination and integration of tasks Centralized control and monitoring of TMT Facilitated by the direct interactions and internal and external connections of TMT Ad‐hoc multifunctional project teams Agility to adapt product and service to meet customer requirements quickly Challenges in ensuring employees understand tasks required – Formal (training) informal (interactions) mechanisms Illustrative quote from interviews: *… converting order to business is a huge challenge. I am involved in all activities. Attention to detail in the design stage is an issue among Indian IT professionals in general. They lack clarity on the actual task. I think it's an issue with the training they received, so we train and prepare them to deal with foreign clients*. (CEO, Case 1)
**Young‐growth phase (3 cases)**	**Scaling‐up firms** **Average age**: 6 years Range: 5–7 **Size**: Micro and small firms Employees (Avg.) = 48 – Range: 25–62 Turnover (Avg.) = $1.6 m – Range: $0.2–$2.5 m **Domain**:Diverse‐IoT, AI, e‐commerce, software architecture and service	**TMT**: TMT‐ED and TMT‐NED (advisors) Average size: 4 Heterogeneous TMT‐level SC and HC – TMT‐EDs with diverse expertise and experience – Experienced industry experts as advisory directors (TMT‐NED) – Confident in their expertise – Leverage network relationships to enhance short and long‐term performance **Role**:Early phase of differentiating TMT‐NEDs from TMT‐EDs Delegating functional‐level responsibilities to functional managers Monitoring and supervising managers and helping in operational decision‐making Illustrative quote from interviews: *We had appointed senior executive managers to lead our functional activities when we moved to the scaling‐up phase. We wanted to bring professionalism and expertise. As the CEO, I am mainly involved in strategic activities now… since we are in the transition phase, other founder directors are still involved in the day to day operations but mainly help the newly appointed functional managers. We were mainly planning to give all operational responsibilities to them*. (CEO, Case 4)	**Goal**: Achieve cost efficiency and aiming to achieve effectiveness through specialization **Key tasks**: Differentiated mainly based on functional expertise Continuous adaptation and non‐repetitive task designs **Structure**: Organizational design is in an evolutionary phase – Moved from an amorphous informal structure to a centralized, semi‐formal and hierarchical organizational architecture – Informal project teams – TMT roles, responsibilities and expectations are clearly defined Illustrative quote from interviews: *We were in the process of formalizing our organizational activities to standardize and professionalize activities. As the first step, we formed different functional departments… we outsourced non‐technological functions such as finance and marketing. We have a more formalized approach with a much clearer delegation of responsibilities and authorities. Although we have small project teams with diverse expertise from different functional groups to deal with specific industries, we don't have a clear and formal work structure at the team level. When we get an order, we* [TMT] *find out how we can do that, who all should be in the team* … *we undertake the work if we think we can. We want to be more formal and specialized* … *We wanted to develop our own product*. (Case 4, CEO)	Centralized, hierarchical and semi‐formalized approach to coordinate and integrate the organizational tasks Direct TMT monitoring and supervision Task implementation through temporary multifunctional project teams Direct involvement of functional directors and TMT in coordinating the multifunctional project teams Illustrative quote from interviews: *We now have both formal and informal mechanisms. For example, our founder directors monitor the performance of our functional managers. The roles and responsibilities of functional departments and their heads are clearly defined now. Directors who are responsible for each function also know their responsibilities. Both functional managers and directors are involved in monitoring and supporting the project teams. They are mainly formed for specific purposes but are usually represented by people from different functional departments*. (TMT‐ED, Case 3)
**Advanced‐growth phase (2 cases)**	**Firms with stable market position** **Average age**: 15.5 years – Range: 11–20 **Size**: Medium‐sized firms Employees (Avg.) = 301 – Range: 252– 350 Turnover (Avg.) = $27 m – Range: $25–$30 **Domain**:Focused – cybersecurity and security services	**TMT**: TMT‐EDs and TMT‐NEDs Homogeneous TMT‐level HC and SC – Focused on core technological domains specialization – Industry‐specific experience and network – Organizational understanding and connection *Illustrative quote from interviews*: *Networking was our forte, so we started business in the same field. It* [cybersecurity] *is an advanced variation of networking. Since all of us had a networking background, it was easier to adapt or migrate to the cybersecurity or IT security domain*. (Founder Director (TMT‐ED), Case 6) **Role**:TMT‐EDs and TMT‐NEDs dealt with long‐term strategic issues and other fiduciary duties Clear role differentiation between TMTs and senior managers Senior managers’ responsibilities were mainly related to operational issues	**Goal**:Achieving differentiation vis‐à‐vis competitors Continuous improvement/modification of product and services innovation **Key tasks**: Narrow and specialized product and service offerings Tasks differentiated into different functional and divisional units **Structure**: Formal, non‐hierarchical and decentralized/delegated organizational structure Complex structure with functional and geographic differentiation Illustrative quote from interviews: *We have a formal system. It is a delegated structure. We have functional units and geographic units. E.g. Asia pacific is a region. It is slightly complex but the roles and responsibilities are also clear to everyone* … *For example, market research is the responsibility of the regional security business head. They have to work with their team to assess the market and determine how we want to address it. They work closely with the pre‐sales team to do the market research*. (Executive, Case 7)	Formalized coordination approach to deal with the complex organization structure Standardization of tasks with norms and procedures Formal documentation of rules and procedures Decentralized monitoring and support Formal committees liaison roles (TMT) Continuous performance review Temporary multifunctional project teams Illustrative quote from interviews: *Everyone is clear about their roles and responsibilities. It's a routine process. It is clearly documented. We provide proper induction and training to our people. We also continuously monitor and provide support whenever required. Our functional managers interact continuously with each other. We* [directors] *also work closely with the TMT to ensure the coordination of different units. We have a more decentralized coordination and communication mechanism now. The technology allowed us to communicate and monitor the process quickly*. (Director, Case 6)*Our regional head will then need to communicate this to the head office, which will then be aligned with the global strategy. Our pre‐sales head at the head office is a sort of liaison officer to coordinate these activities across different regions*. (Non‐executive, Case 7)
**Mature‐growth phase (1 case)**	**Firms with established market position** **Age**: 18 years **Size**: Medium‐sized firms Employees = 450 Turnover = $46 m **Domain**:Diverse multiple technology domains – AI, ML and digital media	**TMT** Composed of TMT‐EDs and TMT‐NEDs Heterogeneous TMT – Diverse human and social capital – Expertise in multiple technology domains – AI, digital media, software development, machine learning (ML) – Board interlocking provided connections and linkages outside not only their core domain but also in different industries – Confidence in their competence – Risk takers and innovative **Role**: TMT‐NEDs – fiduciary role TMT‐EDs‐ strategic planning and decisions – Growth – Innovation – Geographic expansion – Delegating of authority and responsibility Knowledge disseminator	**Goal**: Achieving differentiation vis‐a‐vis competitors. To be the market leader and innovator Planning to further expand their businesses through collaboration or initial public offerings (IPO). **Key tasks** Diverse tasks – Varied technology specialization – Diverse geographic presence – Innovation is key **Structure** Formal delegated and decentralized structure – Highly flexible – Multiple information channels – regional and functional heads act as boundary spanners Illustrative quotation from interviews: *Although we have a complex structure high several departments, multiple specializations, regional focus, the delegation of authority and decision‐making and lack of hierarchy facilitated quick decisions*. … *this also facilitated efficient information processing*. (Executive, Case 8)	Decentralization and formalization facilitated coordination Clear role specifications Standardized norms, rules and procedures for communication Liaison committees to coordinate the functional regional teams Managers (functional and regional) act as the interface
